# P-1871. Surge Protector: Characterizing the Challenges of Fluctuating OPAT Volume

**DOI:** 10.1093/ofid/ofae631.2032

**Published:** 2025-01-29

**Authors:** Asher J Schranz, Madison Ponder, Michael Swartwood, Alan C Kinlaw, Teresa M Oosterwyk, Angela Perhac, Ashley H Marx, Mary Catherine Bowman, Claire E Farel

**Affiliations:** University of North Carolina, Chapel Hill, NC; University of North Carolina at Chapel Hill, Chapel Hill, NC; University of North Carolina Medical Center, Chapel Hill, North Carolina; University of North Carolina Eshelman School of Pharmacy, Chapel Hill, NC 27599-7573, NC; UNC Medical Center, Chapel Hill, North Carolina; University of North Carolina Medical Center, Chapel Hill, North Carolina; University of North Carolina Medical Center, Chapel Hill, North Carolina; UNC, Chapel Hill, North Carolina; UNC Chapel Hill, Chapel Hill, North Carolina

## Abstract

**Background:**

Outpatient parenteral antimicrobial therapy (OPAT) is often overseen by multidisciplinary teams in infectious diseases (ID) provider groups. OPAT programs serve an important role in health systems to decrease inpatient length of stay and ensure safe transitions of medically complex patients. The optimal composition and staffing needs of OPAT teams are not defined. To gauge challenges facing OPAT program administration and provide insights for those initiating an OPAT program, we described trajectories in patient volume throughout our OPAT program.

**Figure:**
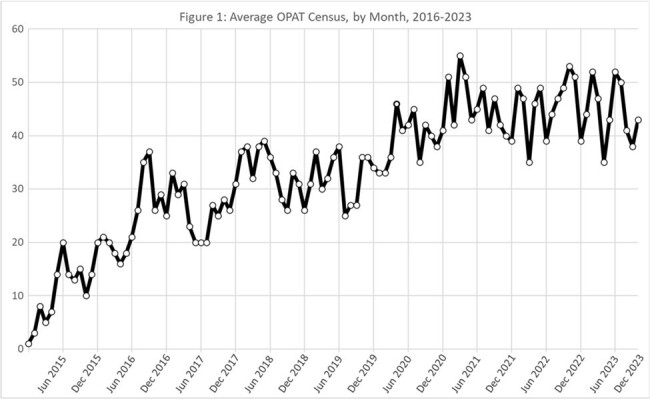

**Methods:**

We included all UNC OPAT courses from inception in 2015 through 2023. The OPAT team mainly provides care for home infusion OPAT, providing lab monitoring, assessment of adverse events, and care coordination. The OPAT team does not cap enrollment and currently accepts patients seen by general ID providers who require >14 days parenteral antimicrobials. OPAT team staffing has varied since inception. It is currently primarily administered by a nurse and a pharmacist, with support from a scheduler and an ID physician. We calculated the average monthly census of patients followed by the OPAT team over time. Census was defined as the number of outpatients followed by the OPAT team at a time. To gain granular detail we also trended the average weekly OPAT census in 2022 and 2023.

**Figure:**
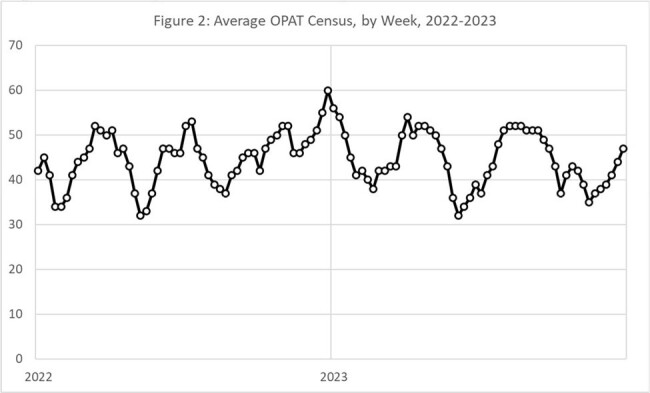

**Results:**

2,878 OPAT courses were cared for by the OPAT team. Median days on OPAT was 35. The most common diagnoses were osteomyelitis (43%), bacteremia (20%) and diabetic foot infection (12%). Average monthly OPAT census overall increased since inception (Fig 1) and demonstrated marked monthly variation across the entire period. In 2022-2023, the weekly census ranged from 32 to 60 patients, with a median weekly census of 45 patients (IQR, 41-50) (Fig 2).

**Conclusion:**

We found our OPAT census underwent frequent, wide fluctuations in volume, which was met with adjustments in task prioritization and approach to accommodate surges. Of note, the OPAT team provides assessment and transition management for referred patients prior to discharge. These patients are not counted in census but are a key area to improve safety and stem readmission. Our results demonstrate that OPAT workload can be volatile and needs robust, expert staffing to meet unpredictable demands and patient needs.

**Disclosures:**

Asher J. Schranz, MD, MPH, UpToDate: I receive fees for authorship of an UpToDate article.

